# Draft Genome Sequences of Strains TAV3 and TAV4 (*Verrucomicrobia*: *Opitutaceae*), Isolated from a Wood-Feeding Termite, and *In Silico* Analysis of Their Polysaccharide-Degrading Enzymes

**DOI:** 10.1128/MRA.01192-19

**Published:** 2020-01-09

**Authors:** Malini Kotak, Jonathan Y. Lin, Jantiya Isanapong, Jorge L. M. Rodrigues

**Affiliations:** a Department of Biology, University of Texas, Arlington, Texas, USA; b Department of Land, Air and Water Resources, University of California, Davis, California, USA; c King Mongkut’s University of Technology North Bangkok, Bangkok, Thailand; d Environmental Genomics and Systems Biology Division, Lawrence Berkeley National Laboratory, Berkeley, California, USA; University of Arizona

## Abstract

Here, we report the high-quality draft genome sequences of *Opitutaceae* sp. strains TAV3 and TAV4, which were isolated from the hindgut of the wood-feeding termite Reticulitermes flavipes. Using a combination of Illumina and PacBio sequencing, we constructed nearly complete assemblies totaling 5.84 and 5.91 Mbp in length for strains TAV3 and TAV4, respectively. In addition, we report an *in silico* analysis of potential lignocellulose-digesting enzymes present in these strains.

## ANNOUNCEMENT

Wood-feeding termites are capable of efficiently digesting lignocellulose with the help of a wide variety of microorganisms residing in their hindgut (1, 2). Previously, five members of the bacterial family *Opitutaceae* (phylum *Verrucomicrobia*) were isolated from the hindgut of the wood-feeding termite Reticulitermes flavipes (3, 4). We used a combination of high-throughput sequencing to reconstruct the genome sequences of two isolates, strains TAV3 and TAV4, from the family *Opitutaceae* (subdivision 4).

The genomes of strains TAV3 and TAV4 were sequenced using Illumina MiSeq and PacBio platforms. For Illumina sequencing, single colonies of strains TAV3 and TAV4 were inoculated in liquid R2B medium from R2 agar plates inoculated from glycerol stocks kept in a −80°C freezer. Liquid cultures were grown at 25°C for 1 week (EMD Chemicals, Billerica, MA), and genomic DNA was isolated using the MasterPure complete DNA and RNA purification kit (Epicentre Biotechnologies, Madison, WI) according to the manufacturer’s instructions. Illumina TruSeq DNA libraries were prepared from paired-end 350-bp inserts and sequenced on the Illumina MiSeq platform with 75-bp paired-end reads. Illumina sequencing yielded 20,683,006 reads for strain TAV3 and 21,234,536 reads for strain TAV4, resulting in >270× sequencing coverage for both strains.

For PacBio sequencing, the strains were cultured as mentioned previously, and the genomic DNA was extracted using the QIAamp DNA minikit (Qiagen, Valencia, CA) according to the manufacturer’s instructions. Extracted DNA was quantified using the Qubit double-stranded DNA (dsDNA) high-sensitivity (HS) assay kit (ThermoFisher, San Jose, CA). The libraries were prepared using a SMRTbell template prep kit (Pacific Biosciences, Menlo Park, CA), and size selection for 20 kb was performed using the BluePippin system (Sage Scientific, Beverly, MA). The genomes of strains TAV3 and TAV4 were sequenced using the Sequel sequencing kit 2.1 and a 600-minute collection time. A total of 943,530 reads (subread *N*_50_, 21,250 bp) were generated for strain TAV3, and a total of 1,093,102 reads (subread *N*_50_, 18,750 bp) were generated for strain TAV4.

Reads were quality checked using FastQC (5), and a hybrid assembly was performed using reads from the Illumina and PacBio runs with Unicycler v0.4.6.0 (6). Default parameters were used for all software unless otherwise specified. The draft genomes were annotated using the NCBI Prokaryotic Genome Annotation Pipeline and Prokka v1.12 (7, 8). The final assembly for strain TAV3 has a total length of 5,844,025 bp containing 4,976 genes predicted from 32 contigs, with an *N*_50_ value of 294,410 bp. Similarly, the TAV4 genome assembly contains 5,914,438 bp with 5,039 genes from 33 contigs, with an *N*_50_ value of 480,638 bp. Both genomes have a GC content of 60.9 mol% and 29 spanned (within scaffold) gaps which comprise 0.049% of the genome sequences.

A comprehensive *in silico* analysis of the carbohydrate-active enzyme (CAZy) profiles identified 433 (8.7% of total) and 431 (8.5% of total) genes in strains TAV3 and TAV4, respectively, as belonging to one of the CAZy families (9). The CAZy annotation identified 235 and 236 genes in TAV3 and TAV4, respectively, as glycoside hydrolases (GHs) belonging to 52 different families (10). TAV3 and TAV4 genomes also contain 83 and 85 glycosyltransferases (GTs), 8 and 7 polysaccharide lyases (PLs), 42 and 40 carbohydrate esterases (CEs), and 61 and 59 carbohydrate-binding modules (CBMs), respectively. Digestion of extracellular cellulose requires the action of secreted enzymes (11). A total of 104 CAZys in each of the strain TAV3 and TAV4 genomes contain signal peptides that suggest extracellular secretion of these proteins. These results show that strains TAV3 and TAV4 have the genomic potential to digest both cellulose and hemicellulose present in plant biomass.

### Data availability.

The whole-genome sequences and annotations for strains TAV3 and TAV4 have been deposited under the DDBJ/ENA/GenBank accession numbers NZ_LXWT00000000 and NZ_LXWU00000000. The NCBI BioProject and BioSample accession numbers for the TAV3 project are PRJNA321366 and SAMN04992812, respectively. The TAV3 raw reads were deposited in the SRA under accession numbers SRR10174322 (PacBio) and SRR3537542 (Illumina). The NCBI BioProject and BioSample accession numbers for the TAV4 project are PRJNA321367 and SAMN04992813, respectively. The TAV4 raw reads were deposited in the SRA under accession numbers SRR10176387 (PacBio) and SRR3537605 (Illumina).
